# Ultrasound Sliding Sign Assessed by Transvaginal or Transrectal Route for Predicting Complete Pouch of Douglas Obliteration in Patients with Ovarian Endometriomas: An Exploratory Retrospective Study

**DOI:** 10.3390/medicina62091791

**Published:** 2026-09-17

**Authors:** Dong Liu, Huanli He, Yuebo Yang, Qingjian Ye

**Affiliations:** Department of Gynecology, The Third Affiliated Hospital of Sun Yat-sen University, Guangzhou 510630, China; liudong8@mail.sysu.edu.cn (D.L.); hehli5@mail.sysu.edu.cn (H.H.)

**Keywords:** ovarian endometriomas, ultrasound sliding sign, pouch of Douglas obliteration, transvaginal ultrasound, transrectal ultrasound, diagnostic accuracy

## Abstract

*Background and Objective*: The ultrasound sliding sign dynamically assesses pouch of Douglas (POD) obliteration in suspected endometriosis. Transvaginal (TVS) and transrectal (TRS) ultrasound are used in patients with and without a self-reported history of vaginal intercourse, respectively, and thus in clinically distinct populations. We evaluated sliding sign performance for predicting surgically confirmed complete POD obliteration in patients with ovarian endometriomas and described findings in route-defined TVS and TRS groups. *Materials and Methods*: This retrospective cohort study enrolled 318 patients with surgically and pathologically confirmed ovarian endometriomas; intraoperative POD status was evaluable in 309. Patients with and without a self-reported history of vaginal intercourse underwent TVS (n = 247) and TRS (n = 71), respectively. A negative sliding sign (absent movement between the uterus and rectum) was the positive index test result for complete POD obliteration. The unadjusted full-cohort diagnostic accuracy analysis was the primary analysis. Because no parous patient underwent TRS (a lack of overlap in examination-route assignment among parous patients), exploratory adjusted analyses were restricted to nulliparous patients, using overlap weighting (principal adjusted analysis) and 1:1 propensity score matching (sensitivity analysis). *Results*: In the primary unadjusted analysis of the full cohort (n = 309), the negative sliding sign showed limited to moderate discriminative ability (sensitivity 0.71, 95% CI 0.63–0.78; specificity 0.60, 95% CI 0.52–0.67; PLR 1.78, 95% CI 1.44–2.20; NLR 0.48, 95% CI 0.36–0.64) and was associated with complete POD obliteration (*p* < 0.001). The between-route sensitivity difference was inconclusive (0.19, 95% CI −0.01 to 0.38). In the exploratory overlap-weighted analysis of nulliparous patients (n = 217), the association remained significant (weighted OR = 2.80, 95% CI 1.17–6.69; *p* = 0.021), without significant sliding sign-by-route interaction (*p* = 0.302); findings were consistent in the matching sensitivity analysis (66 pairs). *Conclusions*: The sliding sign was associated with surgically confirmed complete POD obliteration in patients with ovarian endometriomas, but evidence that this association differed between route-defined groups was inconclusive. Route-specific findings are exploratory because the examination route was determined by sexual history rather than randomization. Whether TRS offers performance comparable to TVS remains undetermined, because the two techniques were not evaluated in the same population and TRS feasibility was not formally assessed.

## 1. Introduction

Endometriosis is a prevalent benign gynecological disorder that primarily affects women of reproductive age. Ovarian endometrioma is one of the most frequently encountered phenotypes of endometriosis. Deep infiltrating endometriosis (DIE) is characterized by endometriotic lesions that penetrate more than 5 mm beneath the peritoneal surface and may involve the uterosacral ligaments, rectovaginal septum, bowel, and pouch of Douglas (POD) [1,2].

Obliteration of the POD represents severe posterior compartment adhesion and may complicate both surgical planning and operative management. Complete POD obliteration may increase operative difficulty and surgical risk and may require more detailed preoperative preparation. Ovarian endometrioma is frequently associated with periadnexal adhesions and may indicate more extensive pelvic adhesive disease, including POD obliteration, although this association is not universal [3]. Therefore, patients with ovarian endometriomas represent a clinically relevant population for evaluating the diagnostic performance of the ultrasound sliding sign.

Ultrasound is widely used for preoperative assessment because it is non-invasive, accessible, and allows real-time dynamic evaluation. The sliding sign evaluates mobility between the uterus and rectum under gentle probe pressure. It was described as a simple and useful non-invasive predictor of POD obliteration [4]. A positive sliding sign indicates free movement between the uterus and rectum, whereas a negative sliding sign indicates absent movement and may suggest POD obliteration or severe posterior compartment adhesions [5].

In routine clinical practice, the ultrasound route is usually determined by self-reported history of vaginal intercourse. TVS is commonly performed in patients with a self-reported history of vaginal intercourse, whereas TRS is used in patients without a self-reported history of vaginal intercourse to avoid potential hymenal injury. However, TVS and TRS are not identical examination routes for assessing the sliding sign. TVS assesses the posterior compartment through the vagina and posterior fornix, whereas TRS evaluates the same region through the rectal wall. Differences in probe position, pressure transmission, viewing angle, and interference from rectal contents may influence assessment of uterorectal mobility.

Importantly, the choice of ultrasound route is not independent of patient characteristics. TVS and TRS are applied to clinically distinct subpopulations defined by self-reported history of vaginal intercourse. These groups may differ in age, parity, pelvic anatomy, prior pregnancy, hormonal exposure, disease duration, previous treatment, or diagnostic delay. Therefore, a retrospective route-defined comparison cannot fully distinguish whether observed differences in diagnostic performance are attributable to the ultrasound route itself or to differences between the underlying patient populations.

Existing evidence regarding the sliding sign remains limited in specific endometriosis phenotypes. Most previous studies included mixed populations with suspected endometriosis or DIE, and few have focused specifically on patients with ovarian endometriomas. Alborzi et al. compared magnetic resonance imaging (MRI), TVS, and TRS in patients with suspected endometriosis and reported the diagnostic performance of these modalities for DIE; however, that study did not specifically evaluate the sliding sign for predicting POD obliteration [6].

This exploratory retrospective study analyzed patients with surgically and pathologically confirmed ovarian endometriomas to evaluate the relationship between the ultrasound sliding sign and surgically confirmed complete POD obliteration. We also described the diagnostic performance of the sliding sign in route-defined TVS and TRS groups under real-world clinical practice. Because the examination route was determined by self-reported history of vaginal intercourse rather than random allocation or paired testing, route-specific findings were interpreted as exploratory and descriptive.

## 2. Materials and Methods

### 2.1. Study Design and Participants

This retrospective cohort study was conducted in accordance with the principles of the Declaration of Helsinki and was approved by the Institutional Review Board of the Third Affiliated Hospital of Sun Yat-sen University (approval no.: II2025-179-01). Owing to the retrospective nature of the study and the use of de-identified clinical data, the requirement for informed consent was waived by the Institutional Review Board.

This study included 318 patients with surgically and pathologically confirmed ovarian endometriomas who underwent laparoscopic surgery between February 2024 and March 2025. A total of 434 potentially eligible patients were assessed during the study period, of whom 116 were excluded because of incomplete documentation (including poor-quality ultrasound images, absent sliding sign documentation, or lack of pathological confirmation of ovarian endometrioma); the individual exclusion reasons were not separately tallied in the retrospective records. No a priori sample size calculation was performed; all consecutive patients meeting the eligibility criteria during the study period were included. Patients were categorized according to the ultrasound examination route used in routine clinical practice. The TRS group consisted of patients without a self-reported history of vaginal intercourse, whereas the TVS group consisted of patients with a self-reported history of vaginal intercourse.

Because the ultrasound route was determined by self-reported history of vaginal intercourse rather than random allocation, the TVS and TRS groups should be regarded as clinically distinct real-world subpopulations rather than interchangeable diagnostic-route groups. Accordingly, route-specific comparisons in this study were considered exploratory and descriptive.

The exclusion criteria were as follows: (i) concurrent genital malignancy; (ii) poor-quality ultrasound images; (iii) lack of pathological confirmation of ovarian endometrioma or absence of a sliding sign assessment.

Patients with missing data for specific variables (intraoperative POD status, dysmenorrhea, height, or weight) were retained in the baseline cohort and were excluded only from the analyses requiring those variables.

This study was reported in accordance with the STARD 2015 and STROBE reporting guidelines.

### 2.2. Data Collection and Examination Methods

#### 2.2.1. Baseline Characteristics

Data collected from electronic medical records included age, height, weight, body mass index (BMI), parity, dysmenorrhea status, preoperative hormonal treatment, maximum endometrioma diameter, operative time, and the revised American Society for Reproductive Medicine (rASRM) score. The rASRM score was used because intraoperative rASRM staging is the institutional routine at our center and was consistently documented in the operative records throughout the study period, whereas the #ENZIAN classification was not routinely recorded. The rASRM score was used solely as a descriptive baseline characteristic and was not included in the propensity score model (Section 2.3), because it is established intraoperatively and partly reflects the outcome being evaluated. The maximum endometrioma diameter was obtained from the preoperative ultrasound report and was defined as the largest documented diameter of the endometrioma on either side. Operative time was defined as the interval from skin incision to completion of skin closure.

#### 2.2.2. Ultrasound Examination

All ultrasound examinations were performed and reported by eight senior gynecologic sonographers (all associate chief physicians and members of the endometriosis team) at our department using a standardized institutional protocol for sliding sign assessment; all reports were reviewed and countersigned by a second senior sonographer. The sonographers had received standardized institutional training in endometriosis ultrasound assessment, including supervised instruction in sliding sign evaluation, before independently reporting examinations. Because this was a retrospective cohort reflecting real-world practice, the sonographer was aware of the clinical indication at the time of examination. Examinations were performed using a Voluson E8 ultrasound system (GE Healthcare, Chicago, IL, USA), with a frequency range of 5–9 MHz for the transvaginal transducer and 5–8 MHz for the transrectal transducer. The sliding sign was assessed dynamically in the sagittal plane by applying gentle probe pressure to evaluate movement between the uterus and the anterior rectal wall: the probe was used to displace the cervix/posterior uterine wall and to compress the anterior rectal wall, and the sign was judged positive only when free sliding between the uterus and rectum was clearly observed in real time. A positive sliding sign indicated free movement between the uterus and rectum, whereas a negative sliding sign indicated absent movement and was considered the index test positive result for predicting complete POD obliteration. For TVS, the probe was placed in the vagina to assess uterorectal mobility through the posterior fornix. For TRS, the probe was inserted rectally to assess mobility between the posterior uterus and the anterior rectal wall. An examination was considered technically adequate only when the uterus, the posterior compartment, and the utero-rectal interface could be adequately visualized; examinations that did not meet this criterion, or in which the sliding sign was unevaluable or not documented, were treated as missing key data and handled according to the exclusion criteria. No standardized bowel preparation protocol was routinely performed before TRS. Sexual activity status was self-reported during routine clinical assessment and was used to determine the ultrasound route.

#### 2.2.3. Surgical Assessment

The exact interval between ultrasound examination and surgery was not systematically recorded in this retrospective dataset; per institutional practice, surgery was scheduled promptly after the ultrasound examination, and the interval did not exceed 1 month in routine practice. Laparoscopic evaluation of POD was performed by a gynecologic surgeon with more than 10 years of experience in laparoscopic surgery for endometriosis. The surgeons were not blinded to the preoperative ultrasound reports, reflecting routine clinical practice (Section 2.4). POD status was classified as (1) no obliteration, (2) partial obliteration, or (3) complete obliteration. Complete obliteration was defined anatomically as complete adhesion of the posterior uterine and cervical surface to the anterior rectal wall, with no identifiable free peritoneal space in the cul-de-sac; partial obliteration was defined as incomplete adhesion with residual free peritoneal space. POD status was assessed intraoperatively in real time and documented in the operative records. Most procedures (266 of 318, 83.6%) were performed by a single senior surgeon (Q.Y.); the remainder were performed by other senior surgeons of the same team using the same institutional definitions. For the binary analysis, “no or partial obliteration” was considered a negative outcome, and “complete obliteration” was considered a positive outcome. This binary endpoint was selected because complete POD obliteration has the clearest implications for surgical planning and operative risk.

### 2.3. Statistical Analysis

Statistical analyses were performed using R software (version 4.5.0) and Python (version 3.13; scikit-learn and statsmodels packages). Continuous variables are presented as medians and interquartile ranges (IQRs), and categorical variables are presented as counts and percentages. Between-group comparisons were performed using the Mann–Whitney U test for continuous variables and the chi-square test or Fisher’s exact test for categorical variables, as appropriate. The propensity score model was specified using clinically relevant preoperative covariates: age, body mass index (BMI), dysmenorrhea status, preoperative hormonal treatment, and maximum endometrioma diameter; to avoid redundancy and collinearity among height, weight, and BMI, BMI was retained as the sole anthropometric covariate. The intraoperatively established rASRM score was deliberately not used as a covariate because it partly reflects the outcome being evaluated. Symptom duration and previous pelvic surgery were not consistently documented in this retrospective dataset and could not be included. Parity was not included in the propensity score model: no parous patient underwent TRS, resulting in a lack of overlap in examination-route assignment among parous patients and quasi-complete separation that no matching or weighting method could resolve. Adjusted analyses were therefore restricted to nulliparous patients. Patients with missing values in any covariate were excluded before propensity score estimation (dysmenorrhea status missing in 15 patients, height or weight in 8, and parity in 1; these missing-data categories did not overlap), leaving 294 patients with complete covariate data, of whom 223 were nulliparous (68 TRS and 155 TVS patients) and eligible for the adjusted analyses. The specified logistic regression model included the examination route as the dependent variable (coded TRS = 0, TVS = 1) and age, BMI, dysmenorrhea status, hormonal treatment, and maximum endometrioma diameter as independent variables. Overlap weighting based on the specified propensity score was used as the principal adjusted exploratory analysis: each TRS patient was weighted by the propensity score and each TVS patient by 1 minus the propensity score, thereby targeting the subpopulation with the greatest covariate overlap. A standardized mean difference (SMD) < 0.1 was considered indicative of adequate balance [7]. Propensity score matching was performed as a sensitivity analysis: one-to-one nearest-neighbor matching without replacement using the specified model, with each TRS patient matched to one TVS patient using a caliper of 0.2 standard deviations of the logit of the propensity score, yielding 66 matched pairs (66 TRS and 66 TVS patients; 132 patients in total).

Diagnostic performance measures, including sensitivity, specificity, Youden index, positive predictive value (PPV), negative predictive value (NPV), positive likelihood ratio (PLR), and negative likelihood ratio (NLR), were calculated for both groups and reported with 95% confidence intervals (CIs). In the unmatched cohort, between-route differences in diagnostic performance were quantified as differences with 95% CIs rather than interpreted solely on the basis of *p* values. The association between the sliding sign and intraoperative POD status was assessed using the chi-square test and the Phi coefficient. In all analyses of the matched cohort, dependence within matched pairs was accounted for as described below.

The unadjusted full-cohort diagnostic accuracy analysis was defined as the primary analysis; overlap weighting was used as the principal adjusted exploratory analysis, and propensity score matching was used as a sensitivity analysis. Overlap-weighted sensitivity, specificity, predictive values, and likelihood ratios were computed from weighted 2 × 2 tables, and 95% CIs and between-route differences were estimated by nonparametric bootstrap (1000 resamples), with the propensity score model re-estimated within each resample. Associations between the sliding sign and complete POD obliteration after weighting were assessed using weighted logistic regression with robust (HC3) standard errors, with and without the sliding sign-by-ultrasound-route interaction term. In the matched sensitivity cohort, dependence within matched pairs was accounted for using logistic generalized estimating equation (GEE) models with robust standard errors and matched subclass as the clustering variable; the model without the interaction term estimated the overall association, and a separate model including the interaction assessed effect modification by route. Post-adjustment PPV and NPV were interpreted cautiously because weighting and matching alter the composition of the analysis population. The association between the sliding sign and POD status in the unadjusted cohort was additionally assessed using the chi-square test and the Phi coefficient. All statistical tests were two-sided, and a *p* value < 0.05 was considered statistically significant.

### 2.4. Strategies to Limit Risk of Bias

To minimize bias, standardized definitions were used for both the index test and reference standard. A positive sliding sign was defined as free movement between the uterus and rectum, whereas a negative sliding sign was defined as absent movement. Complete POD obliteration was defined according to the standardized anatomical criteria described above.

Surgically confirmed POD status was used as the reference standard. Propensity score-based adjustment was performed to reduce measured baseline imbalance between the TVS and TRS groups. However, because the ultrasound route was determined by self-reported history of vaginal intercourse, no adjustment method can eliminate structural confounding or unmeasured differences between groups. Formal blinding of sonographers to the reference standard and of surgeons to the index test results was not implemented, and intraoperative POD status was assessed by surgeons who were aware of the preoperative ultrasound reports; however, both the sliding sign and POD status were assessed and documented as part of routine clinical care using standardized institutional definitions.

Diagnostic accuracy measures were reported with corresponding 95% confidence intervals where available. Residual confounding and other sources of bias, including self-reported history of vaginal intercourse and lack of inter-observer reliability assessment, were considered in the Limitations section.

## 3. Results

### 3.1. Study Population

A total of 318 patients with surgically and pathologically confirmed ovarian endometriomas were included. Among them, 71 patients underwent TRS because they reported no history of vaginal intercourse, and 247 patients underwent TVS because they reported a history of vaginal intercourse.

**Figure 1:**
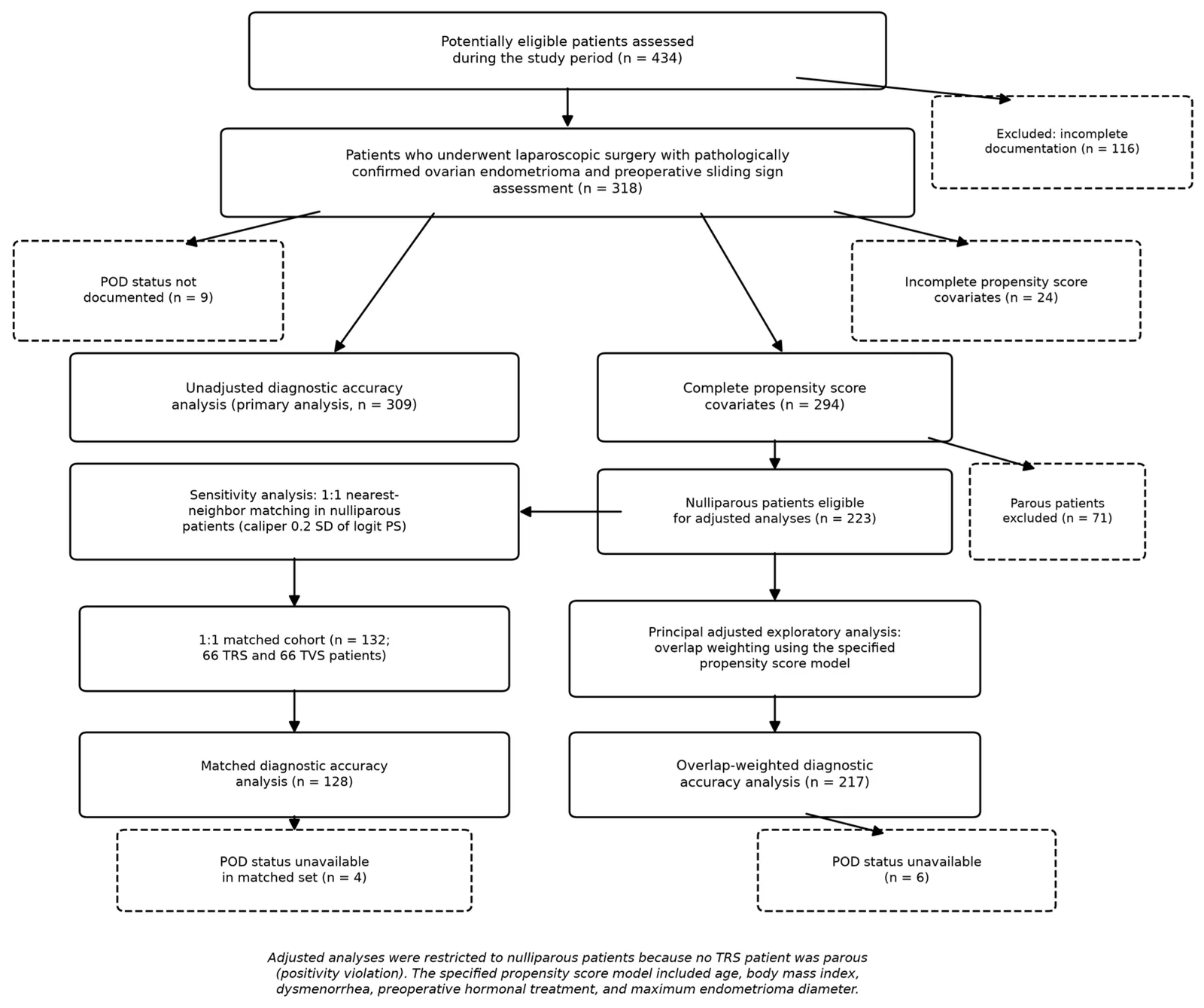
Flow diagram of patient selection and analysis populations. POD, pouch of Douglas; PSM, propensity score matching.

Among the 318 included patients, sliding sign results were available for all patients. Intraoperative POD status was available for 309 patients, who were therefore included in the unadjusted diagnostic accuracy analysis; the remaining 9 patients were excluded from this analysis because intraoperative POD status was not clearly documented in the surgical records. Among the 309 patients with available POD status, 75 (24.3%) had no obliteration, 95 (30.7%) had partial obliteration, and 139 (45.0%) had complete obliteration. The patient flow through the study is summarized in Figure 1. Covariates of the specified propensity score model were incomplete in 24 patients (dysmenorrhea status missing in 15, height or weight in 8, and parity in 1); these patients were excluded from propensity score estimation, which was performed on the remaining 294 patients with complete covariate data. The reasons for missing data were not systematically recorded in this retrospective dataset.

Adjusted analyses were restricted to nulliparous patients because no parous patient underwent TRS (Section 4.3). Among the 294 patients with complete covariate data, 223 were nulliparous (68 TRS and 155 TVS patients), of whom 217 had evaluable intraoperative POD status and were included in the principal adjusted exploratory analysis. In the matching sensitivity analysis, 66 matched pairs (66 TRS and 66 TVS patients; 132 patients in total) were formed; among these, 128 patients had complete data for both the sliding sign and intraoperative POD status (intraoperative POD status was unavailable for 1 TRS and 3 TVS patients). Covariate balance statistics (Table 1) were computed before adjustment, after matching, and after overlap weighting.

**Table 1:** Diagnostic performance of the POD sliding sign before and after propensity score adjustment.

	POD					Statistic (95% CI)						
	No or Partial Obliteration	Total Obliteration	χ^2^	*p*	Phi	Sens	Spec	Youden	PPV	NPV	PLR	NLR
Unadjusted												
Sliding sign of POD (Overall cohort)			30.02	<0.001	0.31	0.71 (0.63–0.78)	0.60 (0.52–0.67)	0.31 (0.21–0.42)	0.59 (0.52–0.66)	0.72 (0.64–0.79)	1.78 (1.44–2.20)	0.48 (0.36–0.64)
Positive	102 (60.00%)	40 (28.78%)										
Negative	68 (40.00%)	99 (71.22%)										
Sliding sign of POD (TRS group)			1.91	0.167	0.17	0.57 (0.39–0.73)	0.60 (0.45–0.74)	0.17 (−0.07–0.40)	0.52 (0.35–0.68)	0.65 (0.49–0.78)	1.42 (0.87–2.32)	0.72 (0.45–1.17)
Positive	24 (60.00%)	13 (43.33%)										
Negative	16 (40.00%)	17 (56.67%)										
Sliding sign of POD (TVS group)			29.87	<0.001	0.35	0.75 (0.66–0.82)	0.60 (0.51–0.68)	0.35 (0.24–0.47)	0.61 (0.53–0.69)	0.74 (0.65–0.82)	1.88 (1.48–2.38)	0.41 (0.29–0.59)
Positive	78 (60.00%)	27 (24.77%)										
Negative	52 (40.00%)	82 (75.23%)										
After overlap weighting												
Sliding sign of POD (Overall cohort)			5.33	0.021		0.67 (0.55–0.78)	0.58 (0.49–0.68)	0.25 (0.10–0.39)	0.55 (0.44–0.65)	0.70 (0.59–0.80)	1.60 (1.20–2.15)	0.57 (0.38–0.82)
Positive	25.4 (66.7%)	21.3 (41.7%)										
Negative	12.7 (33.3%)	29.8 (58.3%)										
Sliding sign of POD (TRS group)			1.13	0.287		0.57 (0.39–0.74)	0.59 (0.44–0.75)	0.16 (−0.08–0.39)	0.55 (0.37–0.72)	0.61 (0.44–0.77)	1.39 (0.84–2.44)	0.73 (0.43–1.17)
Positive	12.0 (57.1%)	9.9 (41.1%)										
Negative	9.0 (42.9%)	14.2 (58.9%)										
Sliding sign of POD (TVS group)			5.18	0.023		0.78 (0.67–0.89)	0.58 (0.47–0.68)	0.36 (0.21–0.49)	0.54 (0.43–0.66)	0.81 (0.71–0.90)	1.86 (1.42–2.48)	0.38 (0.20–0.59)
Positive	13.5 (78.5%)	11.3 (42.0%)										
Negative	3.7 (21.5%)	15.6 (58.0%)										

POD, Pouch of Douglas; Phi, Phi correlation coefficient; Sens, sensitivity; Spec, specificity; PPV, positive predictive value; NPV, negative predictive value; PLR, positive likelihood ratio; NLR, negative likelihood ratio. Sensitivity, specificity, PPV, NPV, PLR, and NLR are presented with their respective 95% confidence intervals. For the overlap-weighted analyses in nulliparous patients, cell entries are weighted counts with weighted column percentages; the χ^2^ statistic is the robust Wald statistic from the weighted logistic regression, and Phi was not computed.

### 3.2. Baseline Characteristics Before and After Propensity Score Adjustment

Before adjustment, 318 patients were included in the baseline analysis, including 71 in the TRS group and 247 in the TVS group. Patients in the TVS group were older than those in the TRS group, with a median age of 30 years (IQR, 27–35) compared with 27 years (IQR, 24.50–29) in the TRS group (*p* < 0.001). Operative time was also longer in the TVS group than in the TRS group (122 min, IQR, 100–160 vs. 103 min, IQR, 85–127; *p* = 0.001). Other variables, including height, weight, BMI, dysmenorrhea, rASRM score, surgery level, and surgical side, showed no statistically significant differences between the two groups before adjustment (Table 2).

**Table 2:** Patients’ clinical characteristics and surgical information before propensity score adjustment.

	History of Vaginal Intercourse				
Parameters	No (n = 71)	Yes (n = 247)	All (n = 318)	χ^2^/Z	*p*
Age, years	27 (24.50, 29)	30 (27, 35)	29 (26, 34)	5.59	<0.001
Height, cm	161 (157, 165)	160 (158, 164)	160 (158, 165)	0.77	0.443
Weight, kg	51.5 (46, 60)	52 (47, 57)	52 (47, 57)	0.20	0.843
BMI, kg/m^2^	19.70 (17.90, 22.60)	20.03 (18.69, 21.83)	19.92 (18.59, 21.88)	0.75	0.453
Maximum endometrioma diameter, cm	7.5 (6.2, 8.3)	7.0 (5.7, 8.5)	7.0 (5.9, 8.5)	1.31	0.190
Dysmenorrhea				0.26	0.609
No	17 (24.29%)	66 (28.33%)	83 (27.39%)		
Yes	53 (75.71%)	167 (71.67%)	220 (72.61%)		
Parity				30.21	<0.001
No	71 (100.00%)	164 (66.67%)	235 (74.13%)		
Yes	0 (0.00%)	82 (33.33%)	82 (25.87%)		
Hormonal treatment				0.29	0.591
No	62 (87.32%)	207 (83.81%)	269 (84.59%)		
Yes	9 (12.68%)	40 (16.19%)	49 (15.41%)		
rASRM score	40 (26.5, 72)	49 (29, 74)	47.50 (28, 74)	1.20	0.230
Surgery level				-	0.576
II	1 (1.41%)	2 (0.81%)	3 (0.94%)		
III	35 (49.30%)	111 (44.94%)	146 (45.91%)		
IV	35 (49.30%)	134 (54.25%)	169 (53.14%)		
Surgery time	103 (85, 127)	122 (100, 160)	120 (92.75, 155)	3.18	0.001
Surgical side				3.20	0.074
Single	60 (84.51%)	181 (73.28%)	241 (75.79%)		
Bilateral	11 (15.49%)	66 (26.72%)	77 (24.21%)		

Values are presented as median (IQR) or n (%). Dysmenorrhea data were available for 303 of 318 patients (missing in 15 patients); percentages for dysmenorrhea were calculated using patients with available data as the denominator. Height and weight were both missing in the same eight patients, and parity was missing in 1 patient; 294 patients had complete data for all covariates of the specified propensity score model. For categorical variables tested using Fisher’s exact test that did not exhibit statistical significance, only *p*-values are reported, and the statistical value is denoted by “-”. Surgery level refers to the intraoperative rASRM stage (II–IV); surgical side refers to unilateral versus bilateral ovarian endometriomas as confirmed at surgery.

**Figure 2:**
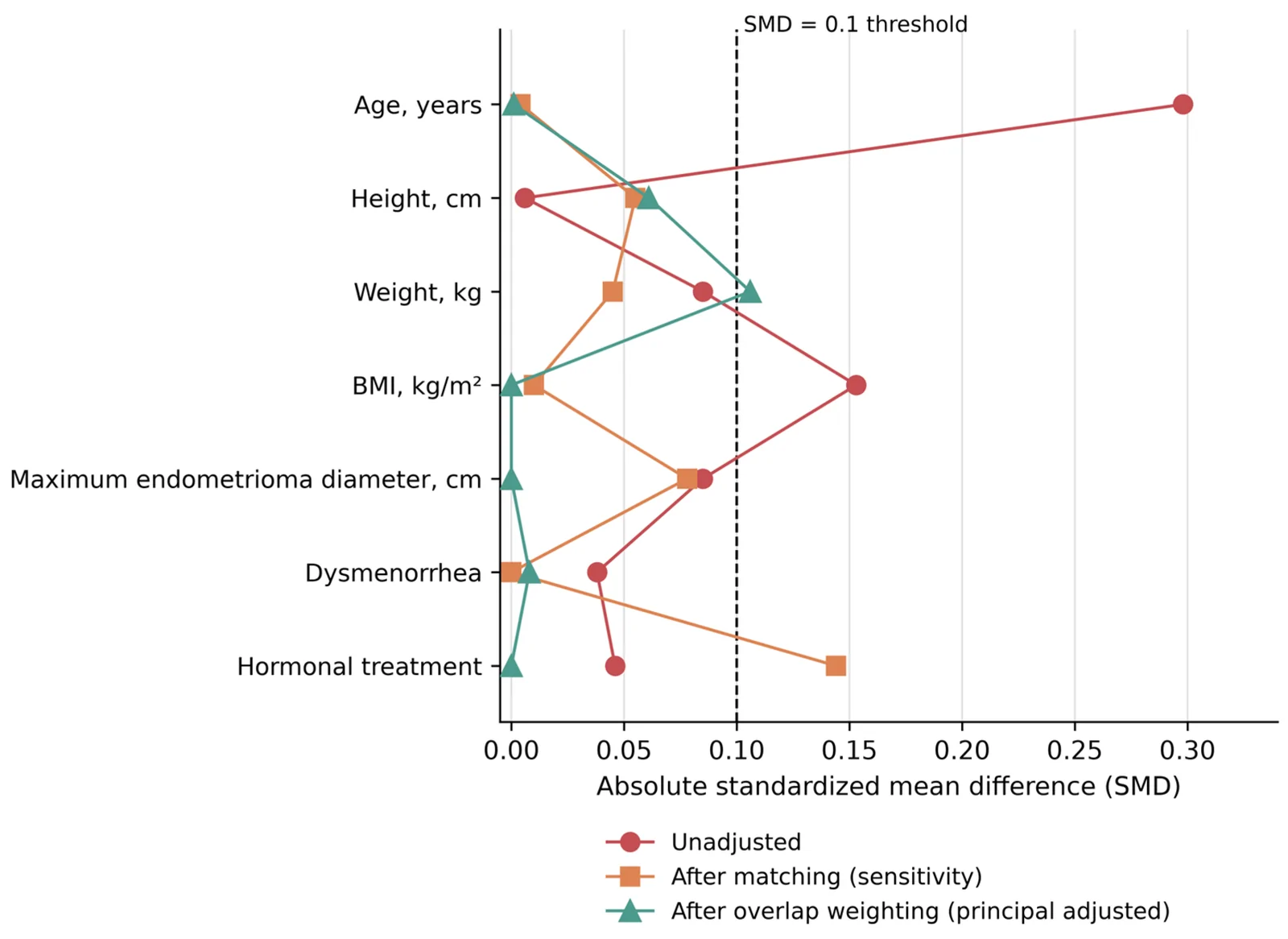
Absolute standardized mean differences (SMDs) of baseline covariates before adjustment, after matching, and after overlap weighting.

In the matched sensitivity cohort (66 TRS and 66 TVS patients), the median age was identical between the two groups (27 years, IQR, 25–29 in both; *p* = 0.629), and no statistically significant differences were observed in height, weight, BMI, dysmenorrhea, hormonal treatment, maximum endometrioma diameter, rASRM score, surgery level, operative time, or surgical side (Table 3). After overlap weighting in nulliparous patients, all covariates included in the propensity score model achieved adequate balance (all absolute SMDs < 0.01; Table 1 and Figure 2); weight, which was not included in the model, remained at an SMD of −0.106, only marginally above the 0.1 threshold in absolute value, and this did not affect the balance of model covariates.

**Table 3:** Patients’ clinical characteristics and surgical information in the matched sensitivity cohort.

	History of Vaginal Intercourse			
	No (n = 66)	Yes (n = 66)	χ^2^/Z	*p*
Age, years	27 (25, 29)	27 (25, 29)	0.48	0.629
Height, cm	160 (157.2, 164.8)	160 (158, 163)	0.15	0.881
Weight, kg	51.8 (47.1, 57.3)	52.5 (47, 58.1)	0.12	0.905
BMI, kg/m^2^	19.5 (17.8, 21.4)	19.8 (18.6, 20.8)	0.37	0.711
Maximum endometrioma diameter, cm	7.6 (6.6, 8.3)	8.0 (6.3, 9.3)	0.65	0.514
Dysmenorrhea			0.00	1.000
No	16 (24.24%)	16 (24.24%)		
Yes	50 (75.76%)	50 (75.76%)		
Hormonal treatment			0.30	0.583
No	57 (86.36%)	60 (90.91%)		
Yes	9 (13.64%)	6 (9.09%)		
rASRM score	39.5 (26.5, 72)	55.5 (29, 77)	1.17	0.242
Surgery level			0.28	0.595
II	0 (0.00%)	2 (3.03%)		
III	34 (51.52%)	29 (43.94%)		
IV	32 (48.48%)	35 (53.03%)		
Surgery time	117.5 (90.5, 150)	117.5 (94.2, 164.8)	0.62	0.533
Surgical side			0.71	0.400
Single	54 (81.82%)	49 (74.24%)		
Bilateral	12 (18.18%)	17 (25.76%)		

Values are presented as median (IQR) or n (%). The matched sensitivity cohort comprised 66 TRS and 66 TVS patients, all with complete covariate data.

No parous patient underwent TRS, resulting in a lack of overlap in examination-route assignment among parous patients; adjusted analyses were therefore restricted to nulliparous patients. In the matched sensitivity cohort, balance was also adequate for all covariates except hormonal treatment, which remained slightly above the threshold (SMD = −0.14).

### 3.3. Unadjusted Diagnostic Performance

In the unadjusted cohort, 309 patients with available intraoperative POD status were included in the primary diagnostic accuracy analysis. In the overall cohort, the sliding sign was significantly associated with complete POD obliteration (χ^2^ = 30.02, *p* < 0.001, Phi = 0.31). The sensitivity and specificity of the negative sliding sign for predicting complete POD obliteration were 0.71 (95% CI, 0.63–0.78) and 0.60 (95% CI, 0.52–0.67), respectively. The Youden index was 0.31 (95% CI, 0.21–0.42), PPV was 0.59 (95% CI, 0.52–0.66), NPV was 0.72 (95% CI, 0.64–0.79), PLR was 1.78 (95% CI, 1.44–2.20), and NLR was 0.48 (95% CI, 0.36–0.64) (Table 4).

**Table 4:** Two-by-two contingency tables of the sliding sign for complete pouch of Douglas obliteration, by cohort and examination route.

Cohort/Route	TP	FN	FP	TN	Total
Unadjusted—Overall	99	40	68	102	309
Unadjusted—TVS	82	27	52	78	239
Unadjusted—TRS	17	13	16	24	70
Matched cohort—Overall	35	19	30	44	128
Matched cohort—TVS	18	6	17	22	63
Matched cohort—TRS	17	13	13	22	65

A negative sliding sign was considered test-positive, and surgically confirmed complete POD obliteration was considered condition-positive. TP, true positive; FN, false negative; FP, false positive; TN, true negative.

In the TRS group, sensitivity and specificity were 0.57 (95% CI, 0.39–0.73) and 0.60 (95% CI, 0.45–0.74), respectively. In the TVS group, sensitivity and specificity were 0.75 (95% CI, 0.66–0.82) and 0.60 (95% CI, 0.51–0.68), respectively. Sensitivity was numerically higher in the TVS group, with a TVS–TRS difference of 0.19 (95% CI, −0.01 to 0.38), whereas the specificity difference was 0.00 (95% CI, −0.18 to 0.18). The 95% CIs for the between-route differences in the other diagnostic performance measures also included zero. The association between the negative sliding sign and complete POD obliteration corresponded to an odds ratio of 1.96 (95% CI, 0.75–5.12) in the TRS group and 4.56 (95% CI, 2.61–7.97) in the TVS group; the estimate was imprecise in the smaller TRS subgroup, and these route-specific estimates are exploratory.

### 3.4. Diagnostic Performance After Propensity Score Adjustment

After overlap weighting in nulliparous patients, 217 patients with evaluable intraoperative POD status contributed to the principal adjusted exploratory analysis. In the weighted overall cohort, sensitivity was 0.67 (95% CI, 0.55–0.78), specificity was 0.58 (95% CI, 0.49–0.68), the Youden index was 0.25 (95% CI, 0.10–0.39), PPV was 0.55 (95% CI, 0.44–0.65), NPV was 0.70 (95% CI, 0.59–0.80), PLR was 1.60 (95% CI, 1.20–2.15), and NLR was 0.57 (95% CI, 0.38–0.82) (Table 4). In the weighted logistic regression, the negative sliding sign remained significantly associated with complete POD obliteration (OR = 2.80, 95% CI, 1.17–6.69; *p* = 0.021). Results were consistent in the matched sensitivity cohort (n = 128): overall sensitivity was 0.65 (95% CI, 0.52–0.76) and specificity was 0.60 (95% CI, 0.48–0.70), and the sliding sign remained associated with complete POD obliteration in the GEE model without the interaction term (OR = 2.70, 95% CI, 1.37–5.34; *p* = 0.004). The corresponding 2 × 2 contingency tables for the matched cohort are provided in Table 5.

**Table 5:** Standardized mean differences in baseline covariates before adjustment, after matching, and after overlap weighting.

Variable	Unadjusted	After Matching (Sensitivity)	After Overlap Weighting (Principal Adjusted)
Age, years	0.298	−0.004	0.001
Height, cm	−0.006	−0.055	−0.061
Weight, kg	−0.085	−0.045	−0.106
BMI, kg/m^2^	−0.153	−0.010	−0.000
Dysmenorrhea	0.038	0.000	0.008
Maximum endometrioma diameter, cm	−0.085	0.078	−0.000
Hormonal treatment	0.046	−0.144	−0.000

SMDs were computed as the difference in means or proportions divided by the pooled standard deviation. The analysis was restricted to nulliparous patients because no parous patient underwent TRS (lack of overlap in examination-route assignment among parous patients); parity was therefore not included in the propensity score model. After overlap weighting, all model covariates had an SMD < 0.01. Height and weight, which were not included in the specified model, are shown for reference (weight showed an SMD of −0.106, marginally above the 0.1 threshold). Overlap weighting achieves balance by reweighting both groups toward the subpopulation with the greatest covariate overlap; it does not imply that the route-defined populations are interchangeable. SMD, standardized mean difference. Covariate balance was assessed in the full matched cohort (n = 132); the matched diagnostic accuracy analysis included the 128 patients with evaluable POD status. An absolute SMD <0.1 was considered indicative of adequate balance.

After overlap weighting, sensitivity was 0.57 (95% CI, 0.39–0.74) in the TRS group and 0.78 (95% CI, 0.67–0.89) in the TVS group, corresponding to a TVS–TRS difference of 0.21 (95% CI, 0.02–0.44). Weighted specificity was 0.59 (95% CI, 0.44–0.75) and 0.58 (95% CI, 0.47–0.68), respectively, with a difference of −0.01 (95% CI, −0.21 to 0.17). In the weighted model that additionally included the interaction term, the sliding sign-by-ultrasound-route interaction was not statistically significant (OR = 2.60, 95% CI, 0.42–16.03; *p* = 0.302), and the matched-cohort GEE analysis yielded a consistent, non-significant interaction (OR = 1.75, 95% CI, 0.37–8.27; *p* = 0.478), providing insufficient evidence that the association between the sliding sign and complete POD obliteration differed between the route-defined groups.

Although adequate covariate balance was achieved after overlap weighting, the adjusted findings should still be interpreted as exploratory because the examination route was not randomly assigned and residual confounding by unmeasured factors (e.g., symptom duration and previous pelvic surgery) cannot be excluded.

## 4. Discussion

### 4.1. Principal Findings

This retrospective study evaluated the diagnostic performance of the ultrasound sliding sign for predicting surgically confirmed complete POD obliteration in patients with ovarian endometriomas. The main finding was that the sliding sign was significantly associated with complete POD obliteration in the overall cohort both before adjustment (χ^2^ = 30.02, *p* < 0.001) and after overlap weighting using the specified propensity score model (weighted OR = 2.80, 95% CI, 1.17–6.69; *p* = 0.021), with consistent results in the matched sensitivity analysis (GEE: OR = 2.70, 95% CI, 1.37–5.34; *p* = 0.004). The unadjusted full-cohort diagnostic accuracy analysis was the primary analysis; overlap weighting was the principal adjusted exploratory analysis, and propensity score matching was a sensitivity analysis. Because the examination route was not randomly assigned, all adjusted results were interpreted cautiously and were not used as the basis for route-specific conclusions. Patients with complete POD obliteration had a higher proportion of negative sliding signs than those with no or partial obliteration.

In route-defined subgroup analyses, this association reached statistical significance in the TVS group but not in the TRS group; however, a significant result in one subgroup and a non-significant result in the other does not, by itself, establish a between-group difference. After overlap weighting, the bootstrap between-route difference in sensitivity was 0.21 (95% CI, 0.02–0.44), whereas the weighted interaction analysis found no conclusive evidence of effect modification by route (OR = 2.60, 95% CI, 0.42–16.03; *p* = 0.302). These analyses address different estimands—a difference in proportions versus a ratio of odds—and, taken together, the evidence for a true route-related difference remains inconclusive. Notably, the study was not powered specifically for the interaction test: the TRS subgroup was small (n = 71), and the interaction estimate was correspondingly imprecise, a range compatible with both negligible and substantial effect modification. The non-significant subgroup and interaction results should therefore be interpreted as absence of evidence rather than evidence of absence, and larger TRS cohorts are needed to resolve whether diagnostic performance truly differs by route.

These findings should be interpreted cautiously, because the comparison between TVS and TRS was based on real-world route-defined groups rather than a head-to-head comparison of interchangeable diagnostic techniques (Section 4.2).

### 4.2. Interpretation of the TVS and TRS Route-Defined Groups

A fundamental limitation of the route-specific analysis is that the ultrasound route was not randomly assigned: in routine clinical practice, TVS was performed in patients with a self-reported history of vaginal intercourse and TRS in patients without a self-reported history of vaginal intercourse, so the two routes were applied to clinically distinct subpopulations rather than comparable or randomly allocated groups. These groups may differ in factors beyond the covariates included in the specified propensity score model, including previous pelvic surgery, pelvic anatomy, symptom duration, symptom profile, and diagnostic delay, and no propensity score-based method can eliminate this structural confounding. Overlap weighting and matching can only balance measured covariates; they cannot establish a causal difference—or equivalence—between TVS and TRS, because the examination route was determined by sexual activity history rather than by randomization or paired testing.

Accordingly, the present study addresses a real-world clinical question: how the sliding sign performs in TVS and TRS groups as they occur in routine clinical practice. It cannot determine whether the ultrasound route itself is responsible for differences in diagnostic performance, nor can it separate the effect of ultrasound route from the effect of sexual activity-related patient characteristics.

### 4.3. Interpretation of Propensity Score-Adjusted Findings

The propensity score model was specified using clinically relevant preoperative covariates: age, body mass index (BMI), dysmenorrhea status, preoperative hormonal treatment, and maximum endometrioma diameter; to avoid redundancy and collinearity among height, weight, and BMI, BMI was retained as the sole anthropometric covariate. Parity was deliberately not included in the model: no parous patient underwent TRS, resulting in a lack of overlap in examination-route assignment among parous patients and quasi-complete separation that no matching or weighting method could resolve. Adjusted analyses were therefore restricted to nulliparous patients, in whom covariate overlap exists. This restriction is the direct consequence of the structural confounding inherent in the route definition: the two routes were applied to clinically distinct populations, and adjusted analyses can only be interpreted within the subpopulation in which both routes are plausible. After overlap weighting, all covariates included in the model achieved adequate balance (all SMDs < 0.1; Table 5 and Figure 2), and the association between the sliding sign and complete POD obliteration remained statistically significant. It should be acknowledged that the matched sensitivity analysis did not achieve adequate balance for all covariates (post-matching SMD for hormonal treatment, −0.14); the overlap-weighted results should therefore be regarded as the principal adjusted findings.

The propensity score model was necessarily restricted to variables that were consistently documented preoperatively. Symptom duration and previous pelvic surgery were not reliably recorded in this retrospective dataset, and CA125 was missing in 51 patients (16%); these variables could not be included. Residual confounding by unmeasured or incompletely recorded factors therefore remains possible.

### 4.4. Comparison with Previous Literature

This study confirmed a significant association between the sliding sign and intraoperative POD status in the overall cohort, which is consistent with previous reports. A systematic review and meta-analysis reported high diagnostic performance of the sliding sign for detecting POD obliteration, supporting its value as a dynamic ultrasound marker [8]. Other studies have also evaluated ultrasound-based assessment of POD obliteration or pelvic adhesions in different clinical settings, including point-of-care ultrasound, structured transvaginal ultrasound assessment, and prediction of pelvic adhesions before gynecologic surgery [9,10,11,12,13]. In addition, the sliding sign has been incorporated into enhanced sonographic approaches, such as sonovaginography or gel sonovaginography, to improve preoperative evaluation of posterior compartment endometriosis [14,15].

The diagnostic performance observed in the present cohort was more moderate. In the full unmatched cohort, the overall sensitivity and specificity were 0.71 (95% CI, 0.63–0.78) and 0.60 (95% CI, 0.52–0.67), respectively, and after overlap weighting the overall sensitivity and specificity were 0.67 (95% CI, 0.55–0.78) and 0.58 (95% CI, 0.49–0.68), respectively, indicating limited to moderate discriminative ability. This discrepancy may be related to differences in study populations. Our cohort focused specifically on patients with ovarian endometriomas, whereas many previous studies included broader populations with suspected endometriosis or higher proportions of DIE. Although ovarian endometriomas are frequently associated with pelvic adhesions, complete POD obliteration is not universal in this population.

With a positive likelihood ratio of 1.78 and a negative likelihood ratio of 0.48, the sliding sign can only modestly shift the level of suspicion and can neither confirm nor exclude complete POD obliteration on its own. Therefore, in patients with ovarian endometriomas, the sliding sign may be useful for raising suspicion of complete POD obliteration, but it should not be used as a standalone diagnostic test.

### 4.5. Findings Specific to Ovarian Endometriomas

Ovarian endometriomas are clinically important because they may coexist with extraovarian endometriosis, pelvic adhesions, and pain-related clinicopathological features [3,16]. In the matched sensitivity cohort, 65 of 128 patients (50.8%) had a negative sliding sign before surgery, and 54 of 128 patients (42.2%) had surgically confirmed complete POD obliteration. These findings indicate that posterior compartment adhesions are common in patients with ovarian endometriomas, but not all patients with ovarian endometriomas have complete POD obliteration.

A negative sliding sign may increase suspicion of complete POD obliteration and may contribute to preoperative planning, but it should not be interpreted in isolation; it should be considered together with clinical symptoms, physical examination, serum markers such as CA125, and other imaging findings.

### 4.6. Route-Specific Considerations for TVS and TRS

TVS and TRS assess the sliding sign through different anatomical routes. TVS places the probe close to the cervix and posterior fornix, allowing relatively direct visualization of the posterior compartment and more direct pressure transmission. TRS evaluates the same region through the rectal wall and may be influenced by rectal gas, fecal material, probe angle, and patient tolerance.

These technical factors may partly contribute to the numerically higher sensitivity and NPV observed in the TVS group after adjustment. However, this interpretation remains speculative because the present study did not directly measure image quality, rectal contents, probe pressure, or operator confidence. In addition, no standardized bowel preparation protocol was routinely performed before TRS, which may have affected visualization.

More importantly, the present study cannot separate route-related technical effects from patient-population differences because the ultrasound route was determined by self-reported history of vaginal intercourse. Therefore, the route-specific findings should not be interpreted as evidence that TVS is intrinsically superior to TRS. A higher weighted sensitivity was observed in the TVS-defined population, but this finding cannot be attributed to the examination route itself. Consistent with this, in a previous diagnostic accuracy study in which both TVS and TRS were performed in the same patients with suspected deep infiltrating endometriosis, the two routes showed broadly comparable accuracy [6]. Despite potential technical limitations, TRS remains clinically important for patients without a self-reported history of vaginal intercourse. Its results should be interpreted in combination with symptoms, biomarkers, and complementary imaging when clinically indicated.

### 4.7. Clinical Implications

For patients with ovarian endometriomas and a self-reported history of vaginal intercourse, TVS remains the standard ultrasound route for dynamic evaluation of the posterior compartment. A negative sliding sign on TVS may indicate increased risk of complete POD obliteration and may inform preoperative surgical planning.

For patients without a self-reported history of vaginal intercourse, TRS remains the applicable ultrasound route in routine practice, but it should not be regarded as a verified equivalent of TVS. However, because the TRS subgroup was relatively small and its estimates were imprecise, TRS findings should be interpreted cautiously. If TRS shows a positive sliding sign but clinical suspicion remains high because of severe dysmenorrhea, bowel symptoms, elevated CA125, or other signs of DIE, additional imaging may be considered according to the overall clinical assessment. MRI-based approaches, including the MRI jelly method and MRI sliding sign, have been explored for assessing cul-de-sac obliteration and rectouterine mobility in pelvic endometriosis [17,18].

Regardless of route, the sliding sign should be considered one component of a multimodal preoperative assessment rather than a standalone diagnostic test.

### 4.8. Strengths and Limitations

This study has several strengths. First, it focused specifically on patients with ovarian endometriomas, a clinically relevant population in whom pelvic adhesions and POD obliteration are important for preoperative assessment. Second, surgically confirmed POD status was used as the reference standard, strengthening the validity of the diagnostic evaluation. Third, the study included both TVS and TRS assessment of the sliding sign and described route-specific diagnostic performance in real-world examination-route groups. Fourth, propensity score-based adjustment, primarily overlap weighting, was used to reduce measured baseline imbalance, and covariate balance was reported transparently.

This study also has several limitations. The most important is the fundamental design limitation discussed in Section 4.2: the ultrasound route was determined by self-reported history of vaginal intercourse, so TVS and TRS were performed in clinically distinct populations rather than in randomly assigned or paired patients, and propensity score-based adjustment cannot fully address the resulting structural confounding.

Second, although restricting the adjusted analyses to nulliparous patients resolved the positivity violation for parity and adequate covariate balance was achieved after overlap weighting, symptom duration and previous pelvic surgery were not consistently documented in this retrospective dataset and could not be included, and CA125 was missing in 16% of patients; residual confounding by these and other unmeasured factors (e.g., detailed obstetric history) cannot be excluded. In addition, because the adjusted analyses apply to the nulliparous subpopulation, their findings may not generalize to parous patients, for whom no TRS data were available. The rASRM score was deliberately excluded because it is established intraoperatively and partly overlaps with the outcome being evaluated.

Several ultrasound-related limitations should also be considered. Although all examinations were performed by eight senior sonographers (all associate chief physicians and members of the endometriosis team) following a standardized protocol, inter-observer reliability of the sliding sign was not formally assessed. Because the sliding sign is a dynamic and partly operator-dependent ultrasound finding, the absence of kappa statistics limits evaluation of reproducibility. In addition, no standardized bowel preparation protocol was routinely performed before TRS, and rectal gas or fecal material may have affected visualization and may partly explain the lower sensitivity observed in the TRS group. The exact interval between ultrasound examination and surgery was not systematically recorded; although surgery was scheduled promptly after ultrasound examination in routine practice, any interval-related change in pelvic findings cannot be excluded. At the same time, because examinations were performed under routine real-world clinical conditions without research-specific standardization, the present results are likely to reflect the diagnostic performance achievable in everyday practice; formal reliability assessment and standardized bowel preparation should be incorporated into future prospective validation studies (Section 4.9).

Finally, this was a single-center retrospective study from South China. Generalizability may be limited by referral patterns, regional characteristics, ultrasound equipment, and operator experience. In addition, because only patients who had been selected for surgery and had pathologically confirmed ovarian endometriomas were included, the reported diagnostic performance applies to this surgical population and should not be directly extrapolated to all patients with suspected endometriosis or to general outpatient populations. Furthermore, the feasibility of TRS (e.g., examination success rate, patient tolerance, and examination time) was not formally evaluated, and examinations of inadequate quality were excluded; therefore, the present data cannot establish the overall feasibility of TRS in unselected patients. In addition, because of the retrospective design, formal blinding of sonographers to the reference standard and of surgeons to the index test results was not implemented, and surgeons were aware of the preoperative ultrasound reports when assessing POD status; however, both the sliding sign and POD status were assessed and documented as part of routine clinical care using standardized institutional definitions, which may have reduced but could not eliminate review bias.

### 4.9. Future Directions

Future studies should use prospective designs with standardized ultrasound protocols, predefined bowel preparation for TRS, structured assessment of image quality, and formal inter-observer reliability evaluation. Where clinically and ethically acceptable, paired assessment of TVS and TRS in the same patients would provide stronger evidence regarding route-specific technical performance. For patients in whom paired testing is not appropriate, prospective multicenter studies with larger TRS cohorts and more comprehensive adjustment for confounders are needed.

Future research should also evaluate ordinal POD status and develop multimodal prediction models integrating sliding sign results, symptoms, biomarkers, MRI findings, cyst characteristics, and surgical history. Emerging plasma and peritoneal fluid markers such as annexin A2 have been reported to be elevated in women with endometriosis, particularly at more advanced stages [19]. Likewise, plasma and peritoneal poly (ADP-ribose) polymerase (PARP) levels have been evaluated as candidate biomarkers in patients with endometriosis, illustrating both the potential value and the current limitations of circulating and peritoneal biomarkers as adjuncts to imaging assessment [20]. Such markers may eventually complement imaging-based assessment, but at present they should be regarded only as possible adjuncts to imaging rather than as specific markers of POD obliteration.

## 5. Conclusions

In surgically treated patients with ovarian endometriomas, a negative ultrasound sliding sign was associated with complete POD obliteration but showed limited-to-moderate diagnostic performance; this association remained statistically significant in the overlap-weighted population. Evidence that this association differed between the route-defined TVS and TRS groups was inconclusive. Because the examination route was determined by self-reported history of vaginal intercourse and residual confounding cannot be excluded, route-specific findings should be considered exploratory. Whether TRS offers performance comparable to TVS remains undetermined, because the two techniques were not evaluated in the same population and TRS feasibility was not formally assessed. The sliding sign should be integrated with other clinical and imaging findings rather than used as a standalone test.

## Data Availability

The datasets generated and analyzed in the current study are available from the corresponding author upon reasonable request.
